# Negative regulation of *HDAC3* transcription by histone acetyltransferase TIP60 in colon cancer

**DOI:** 10.1007/s13258-024-01524-8

**Published:** 2024-05-28

**Authors:** Seong Yun Lee, Junyoung Park, Sang Beom Seo

**Affiliations:** Department of Life Science, College of Natural Science, Chung-Ang University, Seoul, 06974 Republic of Korea

**Keywords:** TIP60, *HDAC3*, Transcription, Colon cancer

## Abstract

**Background:**

Colon cancer is the third most common cancer globally. The expression of histone deacetylase 3 (*HDAC3*) is upregulated, whereas the expression of tat interactive protein, 60 kDa (TIP60) is downregulated in colon cancer. However, the relationship between *HDAC3* and TIP60 in colon cancer has not been clearly elucidated.

**Objective:**

We investigated whether TIP60 could regulate the expression of *HDAC3* and suppress colon cancer cell proliferation.

**Methods:**

RNA sequencing data (GSE108834) showed that *HDAC3* expression was regulated by TIP60. Subsequently, we generated TIP60-knockdown HCT116 cells and examined the expression of *HDAC3* by western blotting and reverse transcription-quantitative polymerase chain reaction (RT-qPCR). We examined the expression pattern of *HDAC3* in various cancers using publicly available datasets. The promoter activity of *HDAC3* was validated using a dual-luciferase assay, and transcription factors binding to *HDAC3* were identified using GeneCards and Promo databases, followed by validation using chromatin immunoprecipitation-quantitative polymerase chain reaction. Cell proliferation and apoptosis were assessed using colony formation assays and fluorescence-activated cell sorting analysis of HCT116 cell lines.

**Results:**

In response to TIP60 knockdown, the expression level and promoter activity of *HDAC3* increased. Conversely, when *HDAC3* was downregulated by overexpression of TIP60, proliferation of HCT116 cells was inhibited and apoptosis was promoted.

**Conclusion:**

TIP60 plays a crucial role in the regulation of *HDAC3* transcription, thereby influencing cell proliferation and apoptosis in colon cancer. Consequently, TIP60 may function as a tumor suppressor by inhibiting *HDAC3* expression in colon cancer cells.

**Supplementary Information:**

The online version contains supplementary material available at 10.1007/s13258-024-01524-8.

## Introduction

Tat-interactive protein, 60 kDa (TIP60) is a member of the MYST family of histone acetyltransferases (HATs) that acetylate histones and non-histone proteins (Avvakumov and Cote 2007; Doyon and Cote 2004). TIP60 is involved in the regulation of various cellular processes, such as DNA damage responses, chromatin remodeling, cell cycle arrest, apoptosis, and gene transcription (Gehlen-Breitbach et al. 2023; Hlubek et al. 2001; Squatrito et al. 2006; Tang et al. 2006). TIP60 can act as both a co-activator and co-repressor due to its transcriptional activity (Hlubek et al. 2001). Generally, TIP60 is known as a co-activator of the transcription of *MYC*, androgen receptor (*AR*), *NF-κB*/*p65*, *Pax6*, and HIV-1 *Tat* (Frank et al. 2003; Gaughan et al. 2002; Kamine et al. 1996; Kim et al. 2012a, 2012b). For example, TIP60 promotes the transcriptional activity of *AR* by binding to and acetylating it. However, TIP60 also bind to genes such as zinc finger E-box-binding protein (*ZEB*) and *ETV6* to perform a co-repressor function or to negatively regulate transcription factors, such as cAMP response element-binding protein (CREB) and p73β (Gavaravarapu and Kamine 2000; Hlubek et al. 2001; Kim et al. 2008; Putnik et al. 2007).

Histone deacetylases (HDACs) are generally known to inhibit gene expression by removing acetylated lysine residues from histones (Berger 2007). Mammalian HDACs are divided into five classes: class I (HDAC1, HDAC2, *HDAC3*, and HDAC8), class IIa (HDAC4, HDAC5, HDAC7, and HDAC9), class IIb (HDAC 6, HDAC 10), class III (sirt1-sirt7), and class IV (HDAC11) (Adhikari et al. 2018). *HDAC3* is a member of class I HDACs and a component of NcoR/SMRT co-repressor complexes (Adhikari et al. 2018; Zhang et al. 2002). *HDAC3* influences cell differentiation, apoptosis, cancer progression, and the cell cycle (Adhikari et al. 2018; Li et al. 2014; Wilson et al. 2006). Previous studies have reported that *HDAC3* is overexpressed in various cancers, such as breast, prostate, and colon cancers (Jeong et al. 2016; Kim et al. 2010; Li et al. 2020). In prostate cancer, *HDAC3* regulates pro-apoptotic genes by decreasing the acetylation of *p53*, a tumor suppressor gene (Jeong et al. 2016). In addition, *HDAC3* reduces p21 activity and apoptosis in colon cancer (Wilson et al. 2006).

Previous studies have demonstrated a relationship between HDACs and TIP60. For instance, HDAC7 binds to the C-terminal region of TIP60, which includes a zinc finger motif (amino acids 261–366), and TIP60 recruits HDAC7 to inhibit the activity of signal transducer and activator of transcription 3 (STAT3) (Xiao et al. 2003). HDAC6 interacts with the TIP60-p400 complex in embryonic stem cells (ESCs), neural stem cells, and cancer cell lines. Specifically, HDAC6 is essential for regulating the target genes of TIP60 in ESCs, and the depletion of HDAC6 leads to decreased cell proliferation (Chen et al. 2013).

In this study, we found that *HDAC3* expression was increased in various cancers and that TIP60 acted as a transcriptional corepressor of *HDAC3* in the colorectal cancer cell line HCT116. We found that cell proliferation was inhibited when the transcriptional activity of *HDAC3* was suppressed by TIP60. In addition, apoptosis increased when the transcriptional level of *HDAC3* was suppressed by TIP60.

## Materials and methods

### Cell culture and transfection

HEK293T cells were obtained from American Type Culture Collection (ATCC; Manassas, VA, USA) and maintained in Dulbecco’s modified Eagle medium (Gibco, Waltham, MA, USA). HCT116 cells were obtained from Korea Cell Line Bank (KCLB; Seoul, Korea) and maintained in RPMI-1640 (Gibco) containing 10% fetal bovine serum (Gibco) and 0.05% penicillin–streptomycin (Welgene) at 37℃ under 5% CO_2_. Transfection of HCT116 and HEK293T cells were performed with polyethyleneimine (PEI; Polysciences, Warrington, PA, USA) at a ratio of 1:3.

### Lentivirus transduction

To produce lentiviral particles, HEK293T cells were co-transfected with plasmids harboring psPAX2, pMD2.G, and the pLKO.1-TRC vector expressing short hairpin (sh)RNAs against TIP60 CDS #1 (5′-TCGAATTGTTTGGGCACTGAT-3′) and CDS #2 (5′-CCTCAATCTCATCAACTACTA-3′). After 48 h of transfection, the supernatants containing the virus particles were collected and used to infect HCT116 cells in the presence of polybrene (8 μg/mL).

### Western blotting analysis

Total cell lysates were prepared using radioimmunoprecipitation assay lysis buffer (50 mM Tris- HCL [pH 8.0], 150 mM NaCl, 0.1% SDS, 0.5% SDC, 1% NP40, 0.5 × protease inhibitor cocktail, and 1 mM EDTA [pH 8.0]). The lysates were agitated for 30 min at 4℃, separated by sodium dodecyl sulfate–polyacrylamide gel electrophoresis (SDS-PAGE), and transferred to nitrocellulose membranes. After transfer, the membranes were incubated at 4℃ overnight with the primary antibodies against TIP60 (sc-166323; dilution 1:5,000), β-actin (sc-47778, dilution 1:1,000) and Bcl-X_L_ (sc-7195, dilution 1:500) from Santa Cruz Biotechnology (Dallas, TX, USA); HDAC2 (ab12169, dilution 1:5,000), *HDAC3* (ab32369; dilution 1:5,000), γ-H2A.X (ab2893, dilution 1:5,000) and PARP1 (ab32138, dilution 1:5,000) from Abcam (Cambridge, UK); HDAC4 (7628S; dilution 1:5,000) from Cell Signaling Technology (Danvers, MA, USA); FLAG (F3165, dilution 1:5,000), HDAC1 (06–720; dilution 1:5,000) and Vinculin (V9131, dilution 1:2,500) from Sigma-Aldrich (Burlington, MA, USA). The membranes were then incubated with the appropriate horseradish-peroxidase-conjugated secondary antibodies and detected using an enhanced chemiluminescence solution.

### Reverse transcription-quantitative polymerase chain reaction (RT-qPCR)

Total RNA was extracted from cells utilizing Tri-RNA Reagent (Favorgen, Pingtung, Taiwan), followed by synthesis of complementary DNA (cDNA). The cDNA was used for mRNA expression analysis by quantitative polymerase chain reaction (qPCR). The RT-qPCR primer sets used in this study are listed in Supplementary Table 1. Dissociation curves were examined after each PCR running. To confirm the amplification of a single product of the appropriate length. The average threshold cycle (CT) and standard error were computed from individual CT values acquired from triplicate reactions at each stage. The normalized average CT value was determined as ΔCT by subtracting the mean CT value of β-actin. The ΔΔCT value was computed as the disparity between the control ΔCT and the corresponding value for each sample. The n-fold alteration in gene expression compared to the expression of the control was determined as 2^−ΔΔCT.^

### Dual-luciferase assay

To conduct the dual luciferase assay, shTIP60 HCT116 cells or FLAG-TIP60 HCT116 cells were co-transfected with a pGL3 basic vector containing the *HDAC3* promoter region and pRL-SV40. After 48 h of transfection, the cells were harvested, and analysis was performed using a dual-luciferase assay system (Promega, Madison, WI, USA).

### Chromatin immunoprecipitation-qPCR (ChIP-qPCR)

The potential transcription factors for *HDAC3* were identified using Promo (Farre et al. 2003) and GeneCards (Fishilevich et al. 2017) databases. Putative binding sites of JUNB within the promoter region of *HDAC3* were analyzed using the JASPAR database (Castro-Mondragon et al. 2022). HCT116 cells were treated with 1% formaldehyde in cell medium for 10 min at 37℃ to induce cross-linking, after which 125 mM glycine was added and cells were incubated for 5 min at room temperature. Following cell lysis in SDS lysis buffer and sonication, immunoprecipitation was performed using specific antibodies. The immunoprecipitates were eluted and subjected to reverse crosslinking. Subsequently, DNA fragments were isolated and purified before PCR amplification. The sequences of the *HDAC3* promoter primers are listed in Supplementary Table 1. The average CT and standard error values were obtained from individual CT values gathered from duplicate reactions at each phase.

### Colony formation assay

HCT116 cells transfected with FLAG, FLAG-TIP60 or FLAG-*HDAC3* were plated in six-well culture dishes at a density of 5 × 10^3^ cells per well. After a 7-d incubation period, surviving colonies were fixed with absolute methanol and stained with 0.01% crystal violet.

### Bioinformatics analysis

Gene ontology (GO) enrichment analyses were conducted using the ShinyGO tool (Ge et al. 2020), based on The Cancer Genome Atlas database accessed using UALCAN (Chandrashekar et al. 2022). The gene expression levels of TIP60 and *HDAC3* in diverse human cancer tissues were identified from the Gene Expression Profiling Interactive Analysis 2 (GEPIA2) database (Tang et al. 2019). Previously published RNA sequencing (RNA-seq) data (GSE108834) (Rajagopalan et al. 2018) were obtained from the Gene Expression Omnibus database.

### Statistical analysis

The results are reported as the mean ± standard error of the mean (SEM) from three independent experiments. Statistical significance (*P*-value) was assessed using GraphPad Prism software by appropriate statistical methods (GraphPad Software, San Diego, CA, USA). The detailed statistical analysis methods are indicated in the figure legends.

## Results

### TIP60 regulated the expression of HDACs in HCT116 cells

**Fig. 1:**
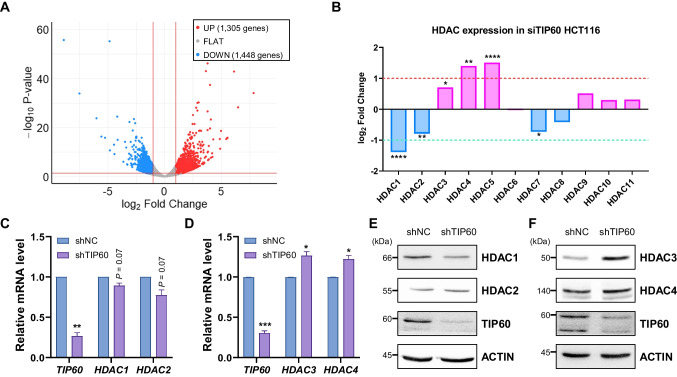
TIP60 regulated the expression of HDACs in HCT116 cells. **A** Volcano plot representing RNA sequencing (RNA-seq) analysis results for GSE108834. Red lines indicate the thresholds for differentially expressed genes (DEGs) (|log_2_ fold change|> 1 and *P* < 0.05). DEG analysis was performed using DESeq2. **B** Expression of histone deacetylases (HDACs) in HCT116 cells transfected with siTIP60 (siTIP60 HCT116 cells) according to the RNA-seq results shown in panel (A). *P < 0.05, **P < 0.01 and ****P < 0.0001. **C** The bar graph represents reverse transcription-quantitative polymerase chain reaction (RT-qPCR) analysis to determine the mRNA levels of *HDAC1* and *HDAC2* in TIP60-knockdown (shTIP60) HCT116 cells. Data are shown as the mean ± standard error of the mean (SEM; n = 3). *P*-values were calculated using paired two-tailed Student’s t-tests. **D** The bar graph represents the RT-qPCR analysis of the mRNA levels of *HDAC3* and *HDAC4* in shTIP60 HCT116 cells. Data are shown as the mean ± SEM (n = 3). *P*-values were calculated using paired two-tailed Student’s t-tests. *P < 0.05 and ***P < 0.001. **E** Western blot analysis to evaluate HDAC1 and HDAC2 protein level in shTIP60 HCT116 cells. **F** Western blot analysis to evaluate *HDAC3* and HDAC4 protein level in shTIP60 HCT116 cells

Previous research has revealed that TIP60 functions as a haplo-insufficient tumor suppressor (Gorrini et al. 2007). To investigate the effect of TIP60 on cancer, we first examined the expression levels of TIP60 in different cancers using the GEPIA2 database. The results revealed a marked decrease in TIP60 expression levels in colon adenocarcinoma (COAD) and rectum adenocarcinoma (READ) (Supplementary Fig. 1A). Furthermore, similar findings were observed in breast invasive carcinoma (BRCA) and uterine corpus endometrial carcinoma (UCEC) (Supplementary Fig. 1B and C). Since reduced expression levels of TIP60 have been observed in colon and rectal cancer, we analyzed previously published RNA-seq data (GSE108834) for the TIP60-depleted colorectal cancer cell line HCT116 (Rajagopalan et al. 2018). TIP60 depletion resulted in the upregulation of 1,305 genes and the downregulation of 1,448 genes (Fig. 1A). Intriguingly, the expression levels of HDAC1, HDAC2, and HDAC7 were downregulated, while those of *HDAC3*, HDAC4, and HDAC5 were upregulated when TIP60 was depleted in HCT116 cells (Fig. 1B). To validate these RNA-seq results, we performed reverse transcription (RT)-qPCR after depleting TIP60 expression in HCT116 cells using an shRNA system. The results revealed that TIP60 knockdown led to a decrease in *HDAC1* and *HDAC2* mRNA levels, which was consistent with the RNA-seq data (Fig. 1C). In addition, *HDAC3* and *HDAC4* levels were increased when TIP60 was depleted in HCT116 cells (Fig. 1D). When we analyzed the protein levels of HDACs in HCT116 cells by western blotting, HDAC3 showed results consistent with those of RNA-seq and RT-qPCR (Fig. 1E and F). Taken together, these findings show that TIP60 is downregulated in various cancers, including colon and rectal cancers, and TIP60 regulates the expression of HDACs, particularly as a transcriptional repressor of *HDAC3* in HCT116 cells.

### *HDAC3* was upregulated in various cancers

**Fig. 2:**
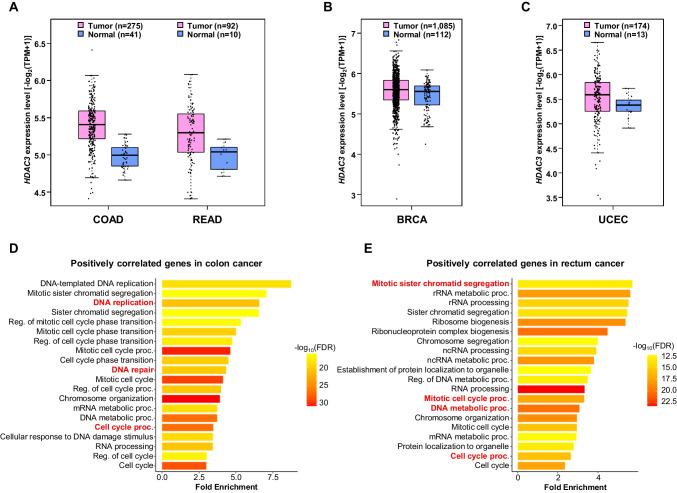
*HDAC3* was upregulated in various cancers. **A** Box plot showing the expression level of *HDAC3* in colon adenocarcinoma (COAD) and rectal adenocarcinoma (READ) compared with corresponding normal tissue. Data were analyzed using the GEPIA2 database. **B** Box plot showing the expression level of *HDAC3* in breast invasive carcinoma (BRCA) compared to the corresponding normal tissue. Data were analyzed using the GEPIA2 database. **C** Box plot showing the expression level of *HDAC3* in uterine corpus endometrial carcinoma (UCEC) compared with the corresponding normal tissue. Data were analyzed using the GEPIA2 database. **D** Gene ontology (GO) analysis result of positively correlated genes with *HDAC3* in colon cancer. The positively correlated genes were identified using UALCAN and GO analysis was performed using ShinyGO. **E** GO analysis result of positively correlated genes with *HDAC3* in rectum cancer. The positively correlated genes were identified using UALCAN and GO analysis was performed using ShinyGO

As we found that the expression of *HDAC3* was markedly increased in TIP60-knockdown cells, we investigated the expression pattern of *HDAC3* in various cancers. The expression of *HDAC3* was upregulated in various cancers, including COAD, READ, BRCA, and UCEC, while the expression of TIP60 was downregulated in these cancers, suggesting the possibility of a transcriptional repressor role of TIP60 in *HDAC3* (Fig. 2A–C and Supplementary Fig. 1A-C). Because the expression of *HDAC3* was greatly upregulated in colon and rectal cancers, we investigated the role of *HDAC3* in these cancers. To this end, we performed GO analysis of genes showing a positive correlation between their expression level and the *HDAC3* expression level in colon and rectal cancers. In colon cancer, genes exhibiting a positive correlation were enriched in pathways related to cell proliferation, including DNA replication, DNA repair, and the cell cycle (Fig. 2D). In rectal cancer, notable enrichment was observed in pathways related to cell growth, including mitotic sister chromatid segregation, nucleic acid metabolic processes, and the cell cycle (Fig. 2E). When we performed GO analysis of BRCA and UCEC using the same method, genes showing a positive expression pattern for *HDAC3* were enriched in pathways associated with mRNA processing, chromatin organization, DNA repair, and the cell cycle (Supplementary Fig. 2A and B). Taken together, the increased expression levels of *HDAC3* in colorectal, breast, and uterine cancers and the GO analysis results of the genes with expression levels positively correlated with those of *HDAC3* suggested that *HDAC3* may play a crucial role in cancer development and malignancy.

### TIP60 acted as a transcriptional repressor of *HDAC3* in HCT116 cells

**Fig. 3:**
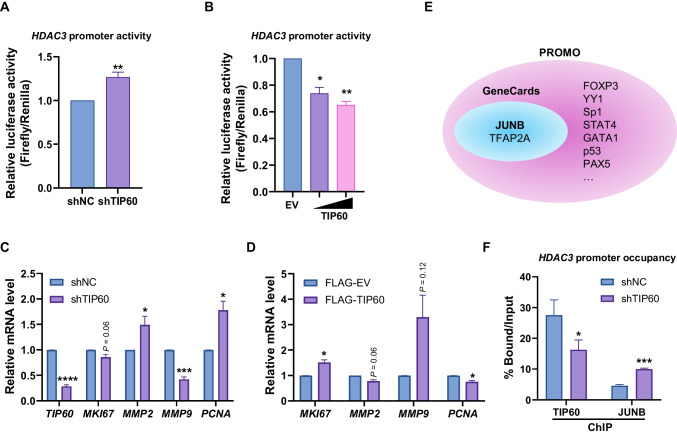
TIP60 acted as a transcriptional repressor of *HDAC3* in HCT116 cells. **A** Dual-luciferase assay to evaluate the promoter activity of *HDAC3* after TIP60 depletion in HCT116 cells. Data are shown as the mean ± SEM (n = 3). *P*-values were calculated using paired two-tailed Student’s t-tests. *P < 0.05. **B** Dual-luciferase assay to evaluate promoter activity of *HDAC3* after TIP60 overexpression in HCT116 cells. Data are shown as the mean ± SEM (n = 3). *P*-values were calculated using one-way analysis of variance (ANOVA) followed by Dunnett’s multiple comparison test. *P < 0.05 and **P < 0.01. **C** The bar graph represents the RT-qPCR analysis of the mRNA levels of HDAC3 target genes in shTIP60 HCT116 cells. Data are shown as the mean ± SEM (n = 3). *P*-values were calculated using paired two-tailed Student’s t-tests. *P < 0.05, ***P < 0.001, and ****P < 0.0001. **D** The bar graph represents the RT-qPCR analysis of the mRNA levels of HDAC3 target genes in TIP60-overexpressing HCT116 cells. Data are shown as the mean ± SEM (n = 3). *P*-values were calculated using paired two-tailed Student’s t-tests. *P < 0.05. **E** Transcription factor analysis was performed to identify the transcription factors that bind to the *HDAC3* promoter region using Promo and GeneCards databases. **F** Chromatin immunoprecipitation-qPCR (ChIP-qPCR) assay to evaluate occupancies of TIP60 and JUNB at the *HDAC3* promoter region in TIP60 knockdown cells. A specific primer set targeting the *HDAC3* promoter region was used. Data are shown as the mean ± SEM (n = 3). *P*-values were calculated using paired two-tailed Student’s t-tests. *P < 0.05 and ***P < 0.001

Since we found that *HDAC3* expression levels were negatively correlated with TIP60 expression levels in HCT116 cells, we hypothesized that TIP60, a transcriptional activator, may act as a non-canonical transcriptional repressor of *HDAC3* in HCT116 cells. To investigate whether TIP60 regulates the transcription of *HDAC3*, we performed a dual-luciferase assay by overexpressing a PGL3 basic vector containing the promoter region of *HDAC3* in HCT116 cells. An increase in *HDAC3* promoter activity was observed in TIP60-knockdown HCT116 cells (Fig. 3A). Additionally, TIP60 overexpression reduced *HDAC3* promoter activity in a dose-dependent manner in HCT116 cells (Fig. 3B). Next, we tested whether HATs other than TIP60 were involved in the transcriptional regulation of *HDAC3*. We performed dual-luciferase assays in HCT116 cells overexpressing EP300 or p300/CBP-associated factor (PCAF). When EP300 or PCAF was overexpressed in HCT116 cells, the promoter activity of *HDAC3* did not change (Supplementary Fig. 3A and B). Conversely, when EP300 or PCAF was simultaneously overexpressed with TIP60, *HDAC3* promoter activity was elevated compared to when only TIP60 was overexpressed (Supplementary Fig. 3A and B). To further investigate whether TIP60 regulates colon cancer progression via HDAC3, we examined its role in modulating HDAC3 target genes. The expression levels of *MKI67*, *MMP2*, *MMP9*, and *PCNA*, HDAC3 target genes associated with cell proliferation and metastasis, were examined (Li et al. 2020). When TIP60 was depleted in HCT116 cells, the mRNA levels of *MKI67* and *MMP9* decreased, whereas those of *MMP2* and *PCNA* increased (Fig. 3C). Conversely, when TIP60 was overexpressed in HCT116 cells, HDAC3 target genes showed opposite expression patterns compared with when TIP60 was depleted (Fig. 3D). To further elucidate the mechanism by which TIP60 regulates the transcription of *HDAC3*, we used GeneCards and Promo databases to identify the transcription factors that bind to the *HDAC3* promoter. Of note, both JUNB and TFAP2A were detected (Fig. 3E). To determine whether the binding of JUNB to the *HDAC3* promoter region was regulated by TIP60, we performed ChIP-qPCR. Increased JUNB occupancy of the *HDAC3* promoter region was detected in TIP60-knockdown HCT116 cells (Fig. 3F). Taken together, these results suggested that, unlike other HATs, such as EP300 and PCAF, TIP60 regulates the recruitment of JUNB to the *HDAC3* promoter region and regulates the transcription of *HDAC3* and its target genes.

### *HDAC3* suppression by TIP60 regulated cell proliferation and apoptosis of HCT116 cells

**Fig. 4:**
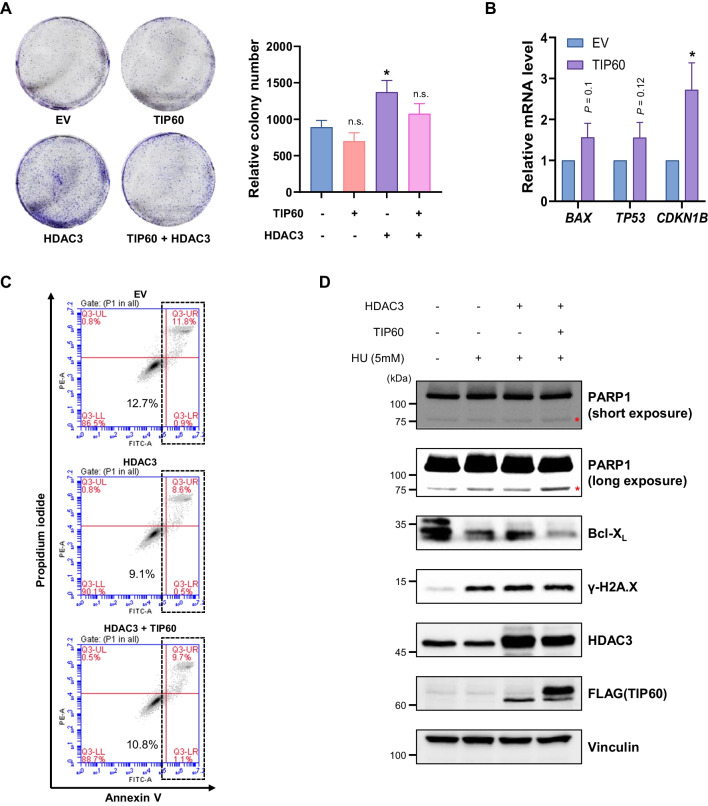
*HDAC3* suppression by TIP60 regulated the proliferation and apoptosis of HCT116 cells. **A** (Left) Representative images of colony formation assay of HCT116 cells in each condition. (Right) The bar graph represents the result of colony formation assay. Data are shown as the mean ± SEM (n = 3). *P*-values were calculated using ANOVA followed by Dunnett’s multiple comparison test. *P < 0.05 and n.s., not significant. **B** The bar graph represents RT-qPCR analysis of the mRNA levels of apoptosis-related genes in TIP60 overexpressing HCT116 cells. Data are shown as the mean ± SEM (n = 3). *P*-values were calculated using paired two-tailed Student’s t-tests. *P < 0.05. **C** The proportion of apoptotic cells were analyzed by flow cytometry. Transfected cells were treated with 5 mM of hydroxyurea (HU) for 32 h to induce apoptosis and stained with propidium iodide (PI) and FITC-Annexin V after trypsinization. **D** Western blot analysis to evaluate apoptosis-related protein levels including PARP1 and Bcl-X_L_ in each condition. Asterisk marks (*) indicate cleaved form of PARP1

Because we found that the transcription of *HDAC3* was repressed by TIP60 and that *HDAC3* had oncogenic activities in colon cancer, we tested whether TIP60-mediated suppression of *HDAC3* affected the proliferation or apoptosis of HCT116 cells. First, we performed colony formation assay to evaluate long-term proliferation of HCT116 cells overexpressing TIP60 or *HDAC3*. As expected, overexpression of TIP60 slightly suppressed proliferation of HCT116 cells (Mattera et al. 2009), whereas overexpression of *HDAC3* promoted proliferation of HCT116 cells; however simultaneous overexpression of TIP60 reversed this effect (Fig. 4A). Next, to investigate whether the expression of apoptosis-related target genes suppressed by *HDAC3* is regulated by TIP60 in HCT116 cells, we performed RT-qPCR (Jiao et al. 2014). When TIP60 was overexpressed, the expression levels of the *CDKN1B*, *TP53*, and *BAX*, known to be suppressed by *HDAC3* and to induce apoptosis, were increased (Fig. 4B). Moreover, we analyzed the effect of *HDAC3* and TIP60 in regulating apoptosis using flow cytometry. To induce apoptosis, hydroxyurea (HU) was treated at concentration of 5 mM for 32 h to transfected HCT116 cells. As expected, *HDAC3* overexpression reduced the proportion of apoptotic cells when HU was treated (Li et al. 2020; Zhang et al. 2017), however, when *HDAC3* was simultaneously overexpressed with TIP60, an increase in the proportion of apoptotic cells was observed (Fig. 4C). In addition, under the same conditions, we checked the protein levels of both apoptosis-related gene and anti-apoptosis-related gene. We observed that the level of the apoptosis marker, cleaved PARP1, increased, while the anti-apoptosis marker Bcl-X_L_ decreased when TIP60 was overexpressed simultaneously with *HDAC3* compared to when *HDAC3* was overexpressed alone. (Fig. 4D). Taken together, these results suggest that TIP60 regulates apoptosis-related target genes of *HDAC3* in colorectal cancer and proliferation by inhibiting *HDAC3* transcription.

## Discussion

Previous studies have suggested that *HDAC3* accelerates the progression of colon and rectal cancers (Li et al. 2020). Other studies have shown that TIP60 plays a role in suppressing colon, rectal, and breast cancers (Rajagopalan et al. 2018; Zhang et al. 2016).

In this study, we identified a relationship between TIP60 and *HDAC3* in the colorectal cancer cell line, HCT116. Initially, we observed a decrease in TIP60 expression levels across various cancers utilizing the GEPIA2 database (Supplementary Fig. 1). Subsequently, we identified *HDAC3* as a notably altered target gene in HCT116 cells transfected with siTIP60 using RNA-seq. Decreased expression levels of *HDAC3* upon TIP60 knockdown were demonstrated by western blotting and RT-qPCR (Fig. 1). Based on these results, we proposed that TIP60 regulates the expression of *HDAC3* in colon cancer cells. Furthermore, through GO analysis, we observed increased expression levels of HDAC3 in various cancers and verified that *HDAC3* influences cell development and malignancies in colon cancer (Fig. 2A–E). Next, using a dual-luciferase assay, we demonstrated that TIP60 suppressed *HDAC3* transcription (Fig. 3A and B). However, the mechanism through which TIP60 regulates the transcription of *HDAC3* remains to be elucidated. Therefore, further research is required to elucidate the mechanism and identify the cofactors that assist TIP60 in suppressing the transcription of *HDAC3*.

Several studies have suggested that TIP60 regulates transcription, either as a co-repressor or a co-activator. TIP60 binds between the *pointed* and *ets* domains of the transcriptional repressor ETV6, resulting in increased nuclear localization, and it acts as a co-repressor to enhance ETV6 activity (Putnik et al. 2007). Furthermore, TIP60 suppresses the transcription of *p73β*, which is structurally similar to p53. While TIP60 is known to function as a co-activator of p53, it interacts with p73β via MDM2, thereby increasing nuclear localization and suppressing transcriptional activity (Kim et al. 2008).

To assess the effect of *HDAC3* inhibition on colon cancer, we examined cell proliferation and apoptosis after inhibiting *HDAC3* in HCT116 cells. These results indicated that the overexpression of TIP60 inhibited the *HDAC3*-mediated proliferation of HCT116 cells. Furthermore, the proportion of apoptotic cells was restored by TIP60 in HCT116 cells (Fig. 4). Therefore, our findings suggest that TIP60 acts as a tumor suppressor by regulating *HDAC3* transcription in colon cancer cells.

### Supplementary Information

Below is the link to the electronic supplementary material.

Supplementary file1 (PDF 543 KB)
