# Supplementary material for: Negative regulation of HDAC3 transcription by histone acetyltransferase TIP60 in colon cancer
Source: Genes Genomics. 2024 May 28;46(7):871–9. doi: 10.1007/s13258-024-01524-8 (PMC11208239; doi:10.1007/s13258-024-01524-8)
Supplement: Supplementary file 1 — Supplementary file1 (PDF 543 KB) [file 13258_2024_1524_MOESM1_ESM.pdf]

Supplementary Information

for

Negative regulation of HDAC3 transcription by histone acetyltransferase TIP60 in colon cancer

by

Seong Yun Lee[1], Junyoung Park[1] and Sang Beom Seo[1,*]

[1]Department of Life Science, College of Natural Sciences, Chung-Ang University, Seoul 06974, Republic of Korea

[*]Corresponding Author: Sang Beom Seo (sangbs@cau.ac.kr)

Supplementary Figure 1. TIP60 is downregulated in various cancers

Supplementary Figure 2. Gene ontology (GO) analysis of gene positively correlated with *HDAC3* in breast and uterine cancer

Supplementary Figure 3. Acetyltransferases EP300 and PCAF had opposite effects on regulating the transcription of HDAC3 compared to TIP60

Supplementary Table 1. List of PCR primer sets used in this study

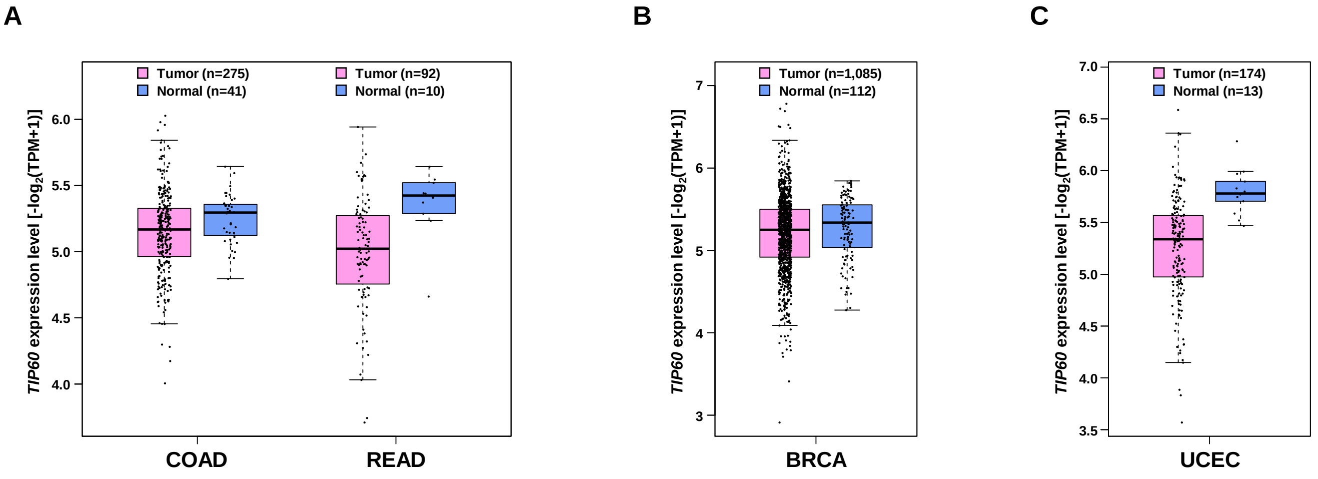

Supplementary Figure 1. TIP60 is downregulated in various cancers

(A) Box plot showing the expression level of *TIP60* in colon adenocarcinoma (COAD) and rectal adenocarcinoma (READ) compared with corresponding normal tissue. Data were analyzed using the GEPIA2 database.

(B) Box plot showing the expression level of *TIP60* in breast invasive carcinoma (BRCA) compared to the corresponding normal tissue. Data were analyzed using the GEPIA2 database.

(C) Box plot showing the expression level of *TIP60* in uterine corpus endometrial carcinoma (UCEC) compared with the corresponding normal tissue. Data were analyzed using the GEPIA2 database.

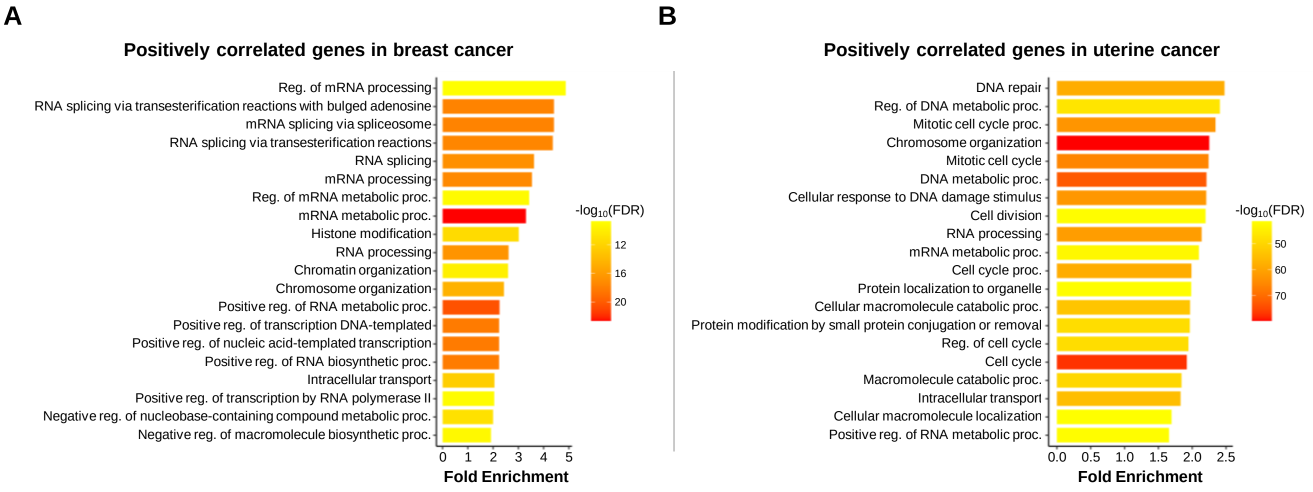

Supplementary Figure 2. Gene ontology (GO) analysis of gene positively correlated with *HDAC3* in breast and uterine cancer

(A) GO analysis result of positively correlated genes with *HDAC3* in breast cancer. The positively correlated genes were identified using UALCAN and GO analysis was performed using ShinyGO.

(B) GO analysis result of positively correlated genes with *HDAC3* in uterine cancer. The positively correlated genes were identified using UALCAN and GO analysis was performed using ShinyGO.

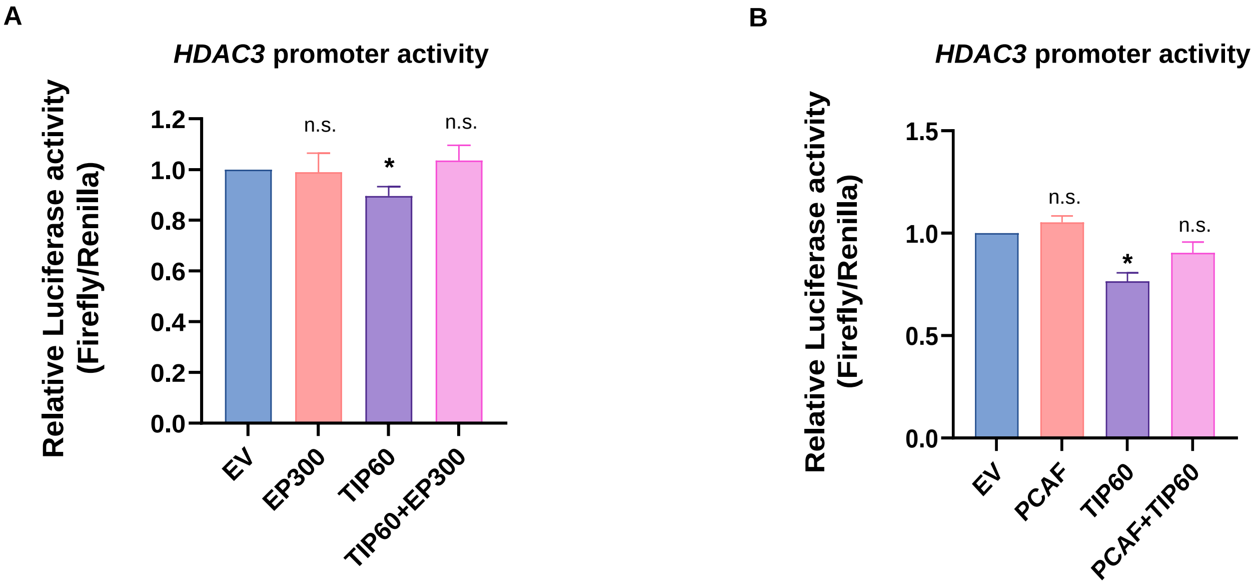

Supplementary Figure 3. Acetyltransferases EP300 and PCAF had opposite effects on regulating the transcription of HDAC3 compared to TIP60

(A) Dual-luciferase assays were used to evaluate the promoter activity of *HDAC3* after overexpressing EP300 and TIP60 in HCT116 cells. Data are shown as the mean ± standard error of the mean (SEM; n = 3). *P*-values were calculated using paired two-tailed Student's t-tests. **P* < 0.05 and n.s., not significant.

(B) Dual-luciferase assays were used to evaluate the promoter activity of *HDAC3* after overexpression of PCAF and TIP60 in HCT116 cells. Data are shown as the mean ± SEM (n = 3). *P*-values were calculated using paired two-tailed Student's t-tests. **P* < 0.05 and n.s., not significant.

Supplementary Table 1. List of PCR primers sets used in this study

Target	Direction	Sequence (5' to 3')	Application
TIP60	Forward	GGAACTCACCACATTGCCTGTC	RT-qPCR
	Reverse	CTCATTGCCTGGAGGATGTCGT	
HDAC1	Forward	GGTCCAAATGCAGGCGATTCCT	RT-qPCR
	Reverse	TCGGAGAACTCTTCCTCACAGG	
HDAC2	Forward	CTCATGCACCTGGTGTCCAGAT	RT-qPCR
	Reverse	GCTATCCGCTTGTCTGATGCTC	
HDAC3	Forward	GAGTTCTGCTCGCGTTACACAG	RT-qPCR
	Reverse	CGTTGACATAGCAGAAGCCAGAG	
HDAC4	Forward	AGGTGAAGCAGGAGCCCATTGA	RT-qPCR
	Reverse	GGTAGTTCCTCAGCTGGTGGAT	
MK167	Forward	GAAAGAGTGGCAACCTGCCTTC	RT-qPCR
	Reverse	GCACCAAGTTTTACTACATCTGCC	
MMP2	Forward	AGCGAGTGGATGCCGCCTTTAA	RT-qPCR
	Reverse	CATTCCAGGCATCTGCGATGAG	
MMP9	Forward	GCCACTACTGTGCCTTTGAGTC	RT-qPCR
	Reverse	CCCTCAGAGAATCGCCAGTACT	
PCNA	Forward	TCTGAGGGCTTCGACACCTA	RT-qPCR
	Reverse	CGCCAAGGTATCCGCGTTAT	
BAX	Forward	TCAGGATGCGTCCACCAAGAAG	RT-qPCR
	Reverse	TGTGTCCACGGCGGCAATCATC	
TP53	Forward	CCTCAGCATCTTATCCGAGTGG	RT-qPCR
	Reverse	TGGATGGTGGTACAGTCAGAGC	
CDKN1B	Forward	GTTTGGAGAGCGGCTGGGTT	RT-qPCR
	Reverse	CAAGCGGAGGAGGGTGGCAAA	
ACTB (β-actin)	Forward	TCCCTGGAGAAGAGCTACGA	RT-qPCR
	Reverse	AGGAAGGAAGGCTGGAAGAG	
JUNB (promoter region)	Forward	GTCCCTCAAAGGTGCGGAAA	ChIP-qPCR
	Reverse	TGGTCTATCCGGTACGCCTG	
